# Corticosterone Methyl Oxidase Type 1 (CMO1) Deficiency Due to CYP11B2 Mutation: Two Case Reports

**DOI:** 10.7759/cureus.39181

**Published:** 2023-05-18

**Authors:** Saad Ur Rehman, Sommayya Aftab, Aamir Naseem, Anjum Saeed, Huma Arshad Cheema

**Affiliations:** 1 Department of Paediatrics and Neonatology, Hameed Latif Hospital, Lahore, PAK; 2 Department of Paediatric Endocrinology and Diabetes, University of Child Health Sciences, The Children's Hospital, Lahore, PAK; 3 Department of Paediatric Endocrinology and Diabetes, Hameed Latif Hospital, Lahore, PAK; 4 Department of Paediatric Gastroenterology, Hepatology and Nutrition, University of Child Health Sciences, The Children's Hospital, Lahore, PAK; 5 Paediatric Gastroenterology, Hepatology and Nutrition, The Children's Hospital and The Institute of Child Health, Lahore, PAK

**Keywords:** novel mutation, aldosterone synthase deficiency, corticosterone methyl oxidase deficiency 1 (cmo1), cyp11b2 gene, isolated aldosterone deficiency

## Abstract

Aldosterone synthase deficiency (ASD) is a rare autosomal recessive condition due to an inactivating mutation in *CYP11B2. *There are two types of ASD depending upon level of defect in aldosterone synthesis, corticosterone methyl oxidase type 1 (CMO 1) and type 2 (CMO 2) deficiency. We are reporting two cases of CMO 1 deficiency presented with failure to thrive. Both cases were born to consanguineous parents and presented at around 17 months and 15 months with complaints of repeated vomiting and failure to thrive. They were found to have persistent hyponatremia, hyperkalemia, low aldosterone level, raised renin levels, normal cortisol and normal 17 hydroxyprogesterone level, suggesting the diagnosis of isolated aldosterone deficiency. Whole exome sequencing revealed that Case 1 is carrying a novel homozygous mutation in *CYP11B2*, c.1391_1393dup p.(Leu464dup) and Case 2 has a homozygous pathogenic variant in *CYP11B2*, c.922T>C p.(Ser308Pro), confirming the diagnosis of CMO 1 deficiency in both cases. After initial stabilization, both cases were started on oral fludrocortisone. They responded well and showed a good catch-up in growth and development. Aldosterone synthase deficiency is a rare condition, but it shall be suspected in infants presented with failure to thrive, hyponatremia and hyperkalemia without pigmentation and virilization.

## Introduction

Aldosterone is the most important mineralocorticoid hormone which is synthesized in the zona glomerulosa layer of the adrenal cortex and is responsible for maintaining intravascular volume and electrolyte balance. Its secretion is regulated by the serum potassium levels, renin angiotensin system and partly by adrenocorticotropic hormone (ACTH) secretion. It acts on mineralocorticoid receptors of different target tissues (kidney, salivary gland, sweat glands and colon) to promote active transport of sodium and excretion of potassium. Isolated aldosterone deficiency is a rare condition due to deficiency of aldosterone synthase, an enzyme encoded by gene *CYP11B2* and is responsible for the final three steps in aldosterone synthesis [1,2].

Aldosterone synthase deficiency (ASD) is a rare autosomal recessive condition that was first reported by Visser and Cost in 1964 [3]. Due to its rare nature its exact incidence is not known. It has two types depending upon level of defect in aldosterone synthesis pathway, corticosterone methyl oxidase type 1 (CMO 1) and type 2 (CMO 2) deficiency. Both types have the same clinical phenotype with varying degrees of severity [4,5]. Children with ASD usually present in infancy with complaints of failure to thrive, repeated vomiting, dehydration, hyponatremia, hyperkalemia, and hypotension [6].

We are reporting two cases with CMO 1 deficiency due to mutation in CYP11B2 from Pakistan, with Case 1 carrying a novel variant of mutation.

## Case presentation

Case 1

A 17-month-old male presented with complaints of failure to thrive, repeated vomiting, and polyuria since birth. He was born full term with a birth weight of 2.5 kg through Cesarean section to consanguineous parents with uneventful antenatal and natal history. Family history was insignificant with three elder healthy siblings. There was no past history of chronic diarrhea, repeated infections, and increased pigmentation. At presentation, he was severely dehydrated with length of 70 cm (-3.60 SD), weight of 5.7 kg (-6.58 SD) and BMI of 11.63 kg/m2 (-6.82 SD). He was pale with loss of subcutaneous fat and reduced muscle mass. He had no pigmentation and had normal-looking male genitalia with bilateral descended testis with volume of 2 ml and pubarche stage 1.

His baseline workup showed low hemoglobin (7g/dl) with microcystic hypochromic anemia and low ferritin level. He had normal blood glucose, liver function test, albumin and bone profile including calcium, magnesium, phosphate, and alkaline phosphatase. His electrolytes showed hyponatremia (Na = 117 mmol/L), hyperkalemia (K+ = 6.8 mmol/L) with raised urea nitrogen (36 mg/dl), normal creatinine (0.21 mg/dl) and metabolic acidosis (pH = 7.2, HCO3 = 10 meq/l). A hormonal profile done at the time of hyponatremia (Na =120 mmol/l) revealed aldosterone levels of 2.49 ng/dl (normal range 1.7 - 23.2 ng/dl), plasma serum renin levels of > 500 ulu/ml (normal 2.8 - 39.9 uiu/ml), serum 17 hydroxyprogesterone levels of 0.23 ng/ml (normal range 0.03 to 1.99 ng/ml), cortisol levels of 6 ug/dl (normal 5.2 - 22.5 ug/dl), ACTH of 13 pg/ml (normal < 46 pg/ml) and aldosterone to renin ratio <1 ng/dl/uiu/ml, suggesting the diagnosis of isolated aldosterone deficiency. The whole exome sequencing revealed that he was carrying a novel homozygous mutation variant in *CYP11B2*, c.1391_1393dup p.(Leu464dup), confirming the diagnosis of autosomal recessive corticosterone methyl oxidase type 1 deficiency. This mutation is an in-frame duplication of three base pairs in exon 8 which causes the duplication of one residue.

He was initially stabilized by rehydrating him with intravenous saline and managing hyperkalemia with IV calcium gluconate and salbutamol nebulization. He started on IV hydrocortisone (100mg/m2/day) along with oral fludrocortisone 200 mcg daily which gradually increased to 400 mcg/day. Once stabilized with normal electrolytes he was discharged on oral fludrocortisone (400 mcg/day). He has been on our follow-up for the last 10 months and is showing good catch-up in growth and development as shown in Table 1. 

**Table 1:** Showing good catch-up of growth and normalization of electrolytes on follow-up of Case 1

Characteristics	At presentation	Follow up (10 months)
Age (months)	17	27
Length/Height (cm) (SD)	70 (-3.60)	79.5 (-1.95)
Weight (kg) (SD)	5.7 (-6.58)	11.4 (-1.14)
BMI (kg/m2) (SD)	11.63 (-6.82)	18.04 (1.42)
HV (cm/y) (SD)	Not available	11.18 (1.57)
Na (meq/l)	117	143
K (meq/l)	6.8	3.4
Fludrocortisone (mcg/day)	400	200

Case 2

A 15-month-old female presented with complaints of repeated vomiting and failure to thrive since birth. She was born full term with a birth weight of 4.5 kg through Cesarean section to consanguineous parents with uneventful antenatal and natal history. Family history of one elder male sibling death at the age of 16 months with the same complaints of repeated vomiting and failure to thrive. She had one elder sister alive and healthy. There was no history of chronic diarrhea, repeated infections, and increased pigmentation. At presentation she was severely dehydrated with length of 65 cm (-4.42 SD), weight of 5 kg (-7.88 SD) and BMI of 11.83 kg/m2 (-6.37 SD). She had no pigmentation and had normal female-looking genitalia with no sign of virilization.

Her baseline workup showed low hemoglobin (6 g/dl) with microcystic hypochromic anemia and low ferritin level. She had normal blood glucose, liver function test, albumin and bone profile including calcium, magnesium, phosphate, and alkaline phosphatase. Her electrolytes showed hyponatremia (Na = 120 mmol/L), hyperkalemia (K+ = 6.7 mmol/L) with raised urea nitrogen (42 mg/dl), normal creatinine (0.20 mg/dl) and metabolic acidosis (pH = 7.2, HCO3 = 12 meq/l). Hormonal profile done at the time of hyponatremia (Na = 120 mmol/l) revealed aldosterone levels of 5.77 ng/dl (normal range 1.7 - 23.2 ng/dl), plasma serum renin levels of > 500 ulu/ml (normal 2.8 - 39.9 uiu/ml), serum 17 hydroxyprogesterone levels of 0.20 ng/ml(normal range 0.03 to 1.99 ng/ml), cortisol levels of 12 ug/dl (normal 5.2 - 22.5 ug/dl), ACTH of 6 pg/ml (normal < 46 pg/ml) and aldosterone to renin ratio < 1 ng/dl/uiu/ml suggesting the diagnosis of isolated aldosterone deficiency. The whole exome sequencing revealed that she was carrying a homozygous mutation in CYP11B2, c.922T>C p.(Ser308Pro) confirming the diagnosis of autosomal recessive corticosterone methyl oxidase type 1 deficiency.

After initial stabilization she was discharged on oral fludrocortisone 400 mcg daily. She has been on our follow-up for the last six months and is showing good catch-up in growth and development as shown in Table 2. 

**Table 2:** Showing good catch-up of growth and normalization of electrolytes on follow-up of Case 2

Characteristics	At presentation	Follow up (6 months)
Age (months)	15	21
Length/Height (cm) (SD)	65 (-4.42)	72 (-3.61)
Weight (kg) (SD)	5 (-7.88)	8.5 (-3.13)
BMI (kg/m2) (SD)	11.83 (-6.37)	16.40 (1.93)
HV (cm/y) (SD)	Not available	14.86 (1.93)
Na (meq/l)	120	142
K (meq/l)	6.7	4.5
Fludrocortisone (mcg/day)	400	400

## Discussion

ASD is a rare autosomal recessive condition due to loss of function mutation in *CYP11B2*, which is located on chromosome 8 and contains nine exons and 503 amino acids. Aldosterone synthase enzyme catalyzes the final three steps of aldosterone synthesis i.e., 11-β hydroxylation of 11-deoxycorticosterone to corticosterone, 18-hydroxylation of corticosterone to 18-hydroxycorticosterone and 18-oxidation 18-hydroxycorticosterone to aldosterone. So, deficiency of this enzyme results in isolated aldosterone deficiency. There are two types of ASD depending upon which step in aldosterone synthesis was defective. In CMO 1 deficiency, also called aldosterone synthase deficiency type 1 (ASD 1), there is a defect in 18-hydroxylation of corticosterone to 18-hydroxycorticosterone leading to normal to low levels of 18-hydroxycortisterone level and low aldosterone level. However, CMO 2 deficiency, also called aldosterone synthase deficiency type 2 (ASD 2), results from defective oxidation of 18-hydroxycorticosterone to aldosterone resulting in high 18-hydroxycortisterone level and low aldosterone level. Both CMO 1 and CMO 2 deficiency share the same clinical spectrum, with CMO 2 a milder phenotype than CMO 1 due to high levels of 18-hydroxycorticosterone which possess a bit of mineralocorticoid activity [7].

ASD is a spectrum disorder with varying severity and can present as early as neonatal age to late adulthood (late-onset familial hypoaldosteronism). In children they usually present in infancy with failure to thrive, repeated vomiting, persistent hyponatremia, and hyperkalemia. However, in adulthood they usually present with orthostatic hypotension, hyperkalemia during an episode volume depletion such as acute gastroenteritis [8]. It is very important to differentiate ASD from other causes of aldosterone deficiency especially salt-losing congenital adrenal hyperplasia (CAH). Clinically, salt-losing CAH could be differentiated from ASD by presence of dark pigmentation and ambiguous genitalia, except in males with 21 hydroxylase deficiency who are born with normal male-like genitalia. Biochemically both conditions present with hyponatremia, hyperkalemia, raised renin levels, and undetectable or low aldosterone levels, but in CAH we will find evidence of primary cortisol deficiency without or without androgen excess (raised ACTH, normal or decreased cortisol levels, raised 17 hydroxyprogesterone levels) [1].

To date 40 different mutations in *CYP11B2* have been reported [7]. It is a very rare condition, and our index cases are the first two cases reported from Pakistan. Our Case 1 is carrying a novel homozygous mutation in *CYP11B2*, c.1391_1393dup p.(Leu464dup) which is an in-frame duplication of three base pairs in exon 3 and causes the duplication of one residue. This mutation variant has never been reported before and we are the first ones to report it. However, our Case 2 mutation variant *CYP11B2* c.922T>C p.(Ser308Pro), which causes an amino acid change from Ser to Pro at position 308, has been previously described by Lovas et al., who reported this variant in three affected family members and reported complete lack of activity of the mutant enzyme in this variant [9]. It might be the reason our Case 2 needed a higher dose of fludrocortisone as compared to Case 1.

Early diagnosis and prompt treatment are crucial for a good clinical outcome. All infants need fludrocortisone, but it is observed that symptoms of salt wasting improve with increasing age and therapy can be discontinued in most children when they grow older. This trend of gradual improvement in salt wasting with increasing age is due to two reasons. First, there is decreased expression of mineralocorticoid receptors in newborn kidneys which gradually increases with age leading to increased sensitivity of mineralocorticoids. Secondly, infants’ milk especially mother's feed is low in sodium and as soon as children start taking table food their dietary Na increases [1,7,9,10].

## Conclusions

We are reporting two cases of CMO 1 deficiency from Pakistan with Case 1 carrying a novel mutation in* CYP11B2.* Although it is a rare condition, a high index of suspicion should be maintained in any infant presented with failure to thrive, hyponatremia and hyperkalemia in absence of pigmentation and virilization.
